# FoodWiki: Ontology-Driven Mobile Safe Food Consumption System

**DOI:** 10.1155/2015/475410

**Published:** 2015-06-15

**Authors:** Duygu Çelik

**Affiliations:** Computer Engineering Department, Istanbul Aydin University, 34295 Istanbul, Turkey

## Abstract

An ontology-driven safe food consumption mobile system is considered. Over 3,000 compounds are being added to processed food, with numerous effects on the food: to add color, stabilize, texturize, preserve, sweeten, thicken, add flavor, soften, emulsify, and so forth. According to World Health Organization, governments have lately focused on legislation to reduce such ingredients or compounds in manufactured foods as they may have side effects causing health risks such as heart disease, cancer, diabetes, allergens, and obesity. By supervising what and how much to eat as well as what not to eat, we can maximize a patient's life quality through avoidance of unhealthy ingredients. Smart e-health systems with powerful knowledge bases can provide suggestions of appropriate foods to individuals. Next-generation smart knowledgebase systems will not only include traditional syntactic-based search, which limits the utility of the search results, but will also provide semantics for rich searching. In this paper, performance of concept matching of food ingredients is semantic-based, meaning that it runs its own semantic based rule set to infer meaningful results through the proposed Ontology-Driven Mobile Safe Food Consumption System (FoodWiki).

## 1. Introduction

Food and drink can influence the risks of health conditions such as heart disease, cancer, diabetes, allergies, obesity, and others. Safe food consumption, especially by patients in these risk groups, has become crucial, as these health problems are ranked among the top ten health risks around the world (http://ods.od.nih.gov/factsheets/Folate-HealthProfessional/). By controlling what and how much to eat as well as what not to eat, we can maximize patients' life quality and decrease usage of unhealthy ingredients or compounds such as oxidants. To get safe food consumption under control by patients, smart e-health systems are helpful that have semantic base knowledge base systems and can be easily extended to provide information from additional e-health tools.

A new Web Technology, Semantic Web (SW) [1] or Web 3.0, has been created with an ontology [2] that contains machine-readable (semantic) annotations. In other words, a SW-based word treasure (vocabulary) can be considered as a special form of ontology or sometimes also simply as a set of URIs (Uniform Resource Identifiers) with a described meaning. Recently, many ontological languages have been proposed and standardized such as RDF(S) (http://www.w3.org/RDF/), Web Ontology Language (OWL) (W3C Recommendation, http://www.w3.org/tr/owl-features/), and its new version OWL 2.0 [3]. OWL expresses concepts in ontological form with specific spatial terms and features. In this way, it is possible to adapt heterogeneous information from distributed information systems. Additionally, each concept described in the ontology encapsulates a subset of instance data from the domain of discourse. The ontology knowledge base provides appropriate results in terms of human communication with the information in a machine-understandable format (OWL) or, in contrast, standardized methods are used for data match from the database, with data written in free style, such as the ingredients of food products on the food package. Thus, the SW is used in order to share and integrate information not only in natural language but also by using the associated software so it can be understood, interpreted, and expressed in a way that makes it easier to find the required data using the software.

The ontology in the proposed system describes health risk groups, unhealthy ingredients or compounds in foods, ingredients unsuitable for those in the risk groups, ingredients with side effects depending on patient characteristics, and so on. Therefore, through the SW approach featuring ontology usage, technical personal health systems are based on ontological knowledge management, which is easily extensible to allow adoption of additional e-health tools. The ontology is shared between personal health services and e-health tools that provide interoperation specified by using OWL.

This paper discusses an Ontology-Driven Mobile Safe Food Consumption System (FoodWiki), available through SW technology, which provides easily carried out and understandable suggestions to a member consumer who is searching for an appropriate packaged food product from market shelves, according to his or her health intolerance information. The packaged food products on the market shelves need to be described to the food consumers via smart suggestion systems according to the consumer's food intolerance information.

According to the law, food firms have to report information about their products such as ingredients, additives, and other details to the country's agriculture ministry. Therefore, the database of the country's agriculture ministry keeps a unique International Article Number (EAN) (http://en.wikipedia.org/wiki/International_Article_Number_(EAN)) of each product via QR codes or barcodes that are printed on the packaging of food products during production. Thus, the proposed system investigates some aspects of the product details via QR code or barcode number and then parses concepts from the system ontology to perform suggestion functionalities. The system determines side effects of nutrients and food additives according to the member consumer's health condition, and then it matches those with the nutrients or food additives of a selected product through a series of semantically based search operations using smart devices during shopping from market shelves. In the future, such safe food suggestion systems may provide smart food menus for individuals by accessing annotations of appropriate foods for them according to their personal health and profile information, through the food knowledge base [4–8].

A national study for safe food consumption with Semantic Web Technologies is not available. However, some research projects are conducted at the international area. One of the recent research projects is completed by the Edamam company (https://www.edamam.com/) that is established in the United States. Edamam's goal was to create a comprehensive food knowledge base becoming an authoritative source of cooking information. The researchers wanted to provide this information in an attractive interface allowing users to search by key words and food classifications. But most of such food services were faced with the common challenge of transforming all of the organic and implied knowledge about food into structured data. With data scattered all across the web, Edamam needed web crawling and mining technology to first identify and extract the recipes. Then they needed to semantically analyze the data by identifying entities, parsing the data, extracting the core information, and classifying the results. Since recipes are duplicated in many cases, they needed to identify when two or more recipes were the same and eliminate duplicates. Therefore Edamam used Ontotext Semantic Platform to map ingredients, cooking techniques, and tools to industry databases including the US Department of Agriculture's Standard Reference which provides a list of some 9000 ingredients, including full nutrition information over 140 nutrients (http://www.ontotext.com/customers/edamam-data-mining/).

Thus, the Edamam food ontology (https://www.edamam.com/) (used to classify everything) included recipes, ingredients, nutrition information, measures, allergies, and more. Based on the semantic facts stored in GraphDB, Edamam applied inferencing to derive more data including cooking time, dietary restrictions (e.g., Vegan, Vegetarian, Kosher, etc.), recipe classifications, recipe complexity, nutrition information per serving, and the degree to which the recipe contributes to a balanced diet. The Edamam vision includes using this platform to build recipe healthy eating applications, shopping applications, cooking robots, and smart fridges. The initial release of the project includes two consumer applications. One project is a mobile application by the Sirma Mobile (http://sirmamobile.com/) that is developed for both iPhone and Android apps (https://www.edamam.com/). Second application was a Web Application (https://www.edamam.com/) that presents ingredient lists, dietary classifications, total energy, a bar of the fundamental nutrients, and detailed nutrition information.

The proposed system is also semantic based mobile application that enables consumers to use an interface as a web service to perform FoodWiki transactions both online or offline. But the differences of the proposed system from Edamam's project are as follows: it considers only packaged products, it considers only some diseases (currently only four allergies), it considers only packaged products, it uses the database of the country's agriculture ministry, and it keeps a unique International Article Number (EAN) of each product via QR codes or barcodes that are printed on the packaging of food products. Also, the system uses its own ontology knowledge base that involves four subsections: person, disease, product, and food ingredients/compounds. We assumed the entire ingredients list of products would be kept in a database of the country's agriculture ministry or food companies. Consequently, the aim of the FoodWiki is to help and give instant detailed information to food sensitive people about their consumed nutrients and food items from packaged products while buying a packaged product on market shelves. However, the proposed system does not consider recipes for healthy eating applications, shopping applications, cooking robots, and smart fridges.

The rest of this paper is organized as follows.  Section 2 presents a detailed description of the Food Ontology Knowledge Base (FOKB) used in the proposed system and also similar food ontologies in the literature.  Section 3 presents the working mechanism and uses concept similarity matching techniques and evaluation criteria to assess the algorithm of the system.  Section 4 describes the inference mechanism that makes use of semantic rules, which is able to identify ingredients with side effects according to consumer health conditions before the food product is consumed.  Section 5 presents the implemented mobile application of the proposed system through a case study; then, Section 6 depicts tool and methods used, while Section 7 is dedicated to conclusions.

## 2. Food Ontology Knowledge Base (FOKB)

The system uses its own Food Ontology Knowledge Base (FOKB) that involves four subsections: person, disease, product, and food ingredients/compounds. The person and disease sections have similar contents that describe the general properties of individuals and some nutrition-related human diseases. Currently, the disease section contains only some well-known intolerance/allergy symptoms commonly seen in people such as those associated with lactose, fish, gluten, and egg allergies. The section will be extended in future versions of the FOKB to include other common diseases that may improve in time with the right nutrition, such as heart disease and diabetes.

The current FOKB contains four main classes, 58 subclasses, 15 object type properties and 17 subobject type properties, 12 data type properties, 1530 individuals with annotation type properties, and 210 semantic rules. FOKB starts with a “thing” class that contains four main classes: “diseases,” “person,” “ingredients,” and “product.” The classes contain many subclasses, properties, and individuals that are presented in Tables 1 [Table tab2] [Table tab3] [Table tab4]–5.

The food ingredients/compounds ontology contains many food domain-related concepts, such as food allergens, antioxidants, acidity regulators, food concepts, and food additives, where each part of the ontology contains food domain-based concepts, properties, and relationships. These are depicted in Tables 6 and 7.

Another class of the FOKB is the product class (Table 8) that represents an abstract model of different types of foods, containing nutritional information for foods including the types and amount of nutrients, additives/compounds information, energy information, and the recommended daily intake.

**Figure 1:**
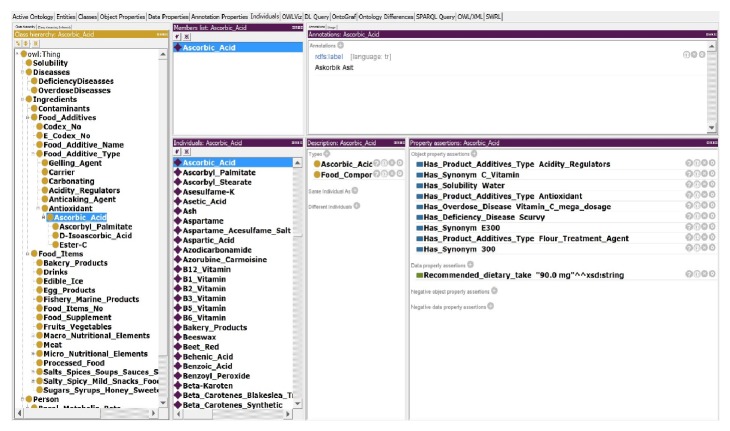
A small portion of the Food Ontology Knowledge Base (FOKB) on the Protégé ontology editor.

Some of the classes, subclasses, and the individual entries of the FOKB are depicted above; they were created using the Protégé ontology editor (http://protege.stanford.edu/download/registered.html#p4.1) [9] (Figure 1). In addition, the ontological structure of the FOKB, or in another words its semantic rule set, was created with Protégé using SWRL (http://www.w3.org/Submission/SWRL/) (Semantic Web Rule Language) [10].

Table 9 depicts a portion of the FOKB in the OWL form that contains almost 1600 semantic declarations about compounds and food additives in the food domain, such as “Acidity_Regulator”, “Anticaking_Agent”, “Auxiliary Components”, “Codex Numbers”, “Antioxidant”, “Calories”, or “Food_Additives”. The “Food_Additives” section contains also various subclasses, such as “Anticaking_Agent”, “Antifoaming_Agent”, or “Antioxidant”. The ontology also contains properties such as “hasAdditives”, “hasAminoAcid”, “hasCalorie”, “hasSynonym”, and others in the food domain. The semantic food contexts are described in FOKB by using OWL 2.0 semantic tags such as *〈*owl:class*〉*, *〈*rdfs:subClassOf*〉*, *〈*owl:DatatypeProperty*〉*, and *〈*owl:ObjectProperty*〉*.

In addition, Table 10 depicts the model used while creating the concepts and properties of the “**#C_Vitamin**” as food additive in OWL 2.0 form, which is shown in Table 9.

As a result, FOKB contains related concepts, properties, and annotations of food knowledge, such as food products, allergy conditions, person profiles, nutrients, food additives, and energy information, formatted in a semantic way. To describe details of the proposed system, a portion of the FOKB is depicted in Table 9 and considered as a case study for the rest of the paper. The class diagram of the FOKB is given in Appendix A of this paper. The working mechanism and mathematical modeling of the system in pseudo code format are discussed in the next section.

## 3. Working Mechanism of the Ontology-Driven Mobile Safe Food Consumption System (FoodWiki)

The Ontology-Driven Mobile Safe Food Consumption System (FoodWiki) is designed based on semantic search, match, and inference techniques. The system will enable customers to use an interface as a web service to perform online matching of intolerance and ingredient concepts. The system application provides authentication of a member consumer who can search to get appropriate food consumption suggestions about the queried food product from the market shelves. The consumers get a report on these queried products that gives information about nutrients contained, fat details, and food additive details such as allergen elements inside the products, according to the individual's health profile in keeping with FoodWiki. In addition, the system gives the consumer an intolerance score according to the involved ingredients and food additives in the chosen product on the market shelves before the individual consumes the product.

**Figure 2:**
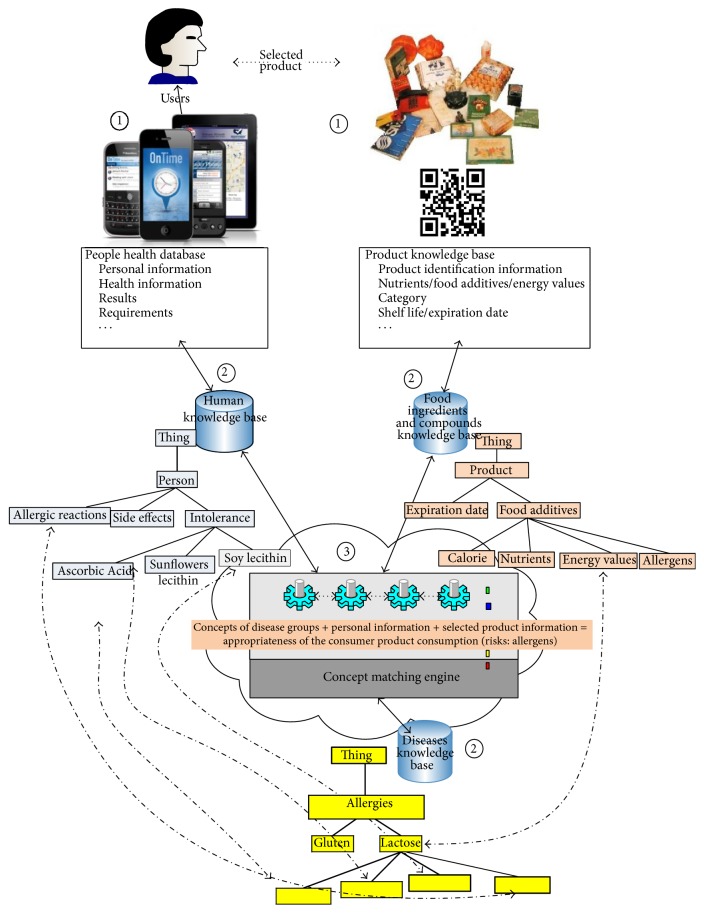
System working mechanism.

User interfaces of the system are designed for ease of use, and they cater to user needs, working in online or offline modes, for use anywhere, anytime. First, the system gets the nutritional information of a selected product from the database according to its unique EAN number (through QR code or barcode number of the product; Step 1, Figure 2). Then, the system starts to search its ontology knowledge bases to retrieve nutritional information on the selected product and side effects of the nutrients according to the consumer's health or disease type (Step 2, Figure 2). In addition to retrieving the nutritional concepts about commercial products from the ontology, then the system compares that with the specific nutritional concepts that have side effects for consumer health (Step 3, Figure 2) through a concept-based matching task. In order to perform this comparison, the system needs to use semantic-based matching and reasoning through the concepts and relationships defined in the FOKB. The semantic matching of retrieved concepts and reasoning through FOKB are depicted as a system mechanism (Figure 2) and performed by a concept matching engine. The concept matching engine performs two sequence tasks: semantic enhancement of concepts as a first step and then concept matching tasks, which are further discussed below.

### 3.1. Semantic Enhancement of the Concept Matching Engine (CME)

Initially, the database of the system keeps the various personal and health data as profile information of a consumer in the* consumer intolerance list* in the food domain. The aim of the semantic enhancement step of the CME is to generate a new list from the terms in the* consumer intolerance list*. Each term of the list relates in the FOKB to various ontology concepts with various additional concepts through meaningful linked relations such as synonymy, “Is_a”, and “has additive type or group”. In addition, the database of the system holds the QR code or barcode numbers with the information on involved food additives and ingredient or nutrient details of products sold on market shelves. The ingredient list of a selected product will be taken from the database of the country's agriculture ministry that keeps a unique International Article Number (EAN) of each produced product as QR codes or barcodes. The codes are printed on the food product packaging during production by the producer food firms. Therefore, the system has two lists as inputs to the CME to perform the semantic enhancement step.

- *Consumer Intolerance List*. It is the list of terms for side effects of nutrients according to a consumer's health or disease type (i.e., allergens, food additives, ingredients, food items, etc.)
- *Selected Product Ingredients List*. It is the list of terms for food additives, ingredients, or nutrients of the selected product.

At the end of this step, the CME generates another list that is called a* semantically enhanced consumer intolerance list* from the* consumer intolerance list*. In order to perform the semantic enhancement task, the system parses appropriate concepts and relationships defined in the food ingredients section of the FOKB. Namely, concepts related to food ingredients or compounds and associated properties are parsed according to the terms in the* consumer intolerance list*.

The reason for using ontologies is that while food companies label their products, they do not use standard concepts for ingredients and namely may use E-codex, abbreviations, or synonyms of the ingredient concepts during the labeling process. In addition, there are many alternative concepts that are used for food ingredients or chemical concepts on food product labels. For example, l-Ascorbic Acid, Vitamin C, Ascorbate, Ascoltin, L(+)-Ascorbic Acid, Ascorbicap, Hybrin, Cevitamic Acid, and L-Ascorbate are also known as Ascorbic Acid.

**Figure 3:**
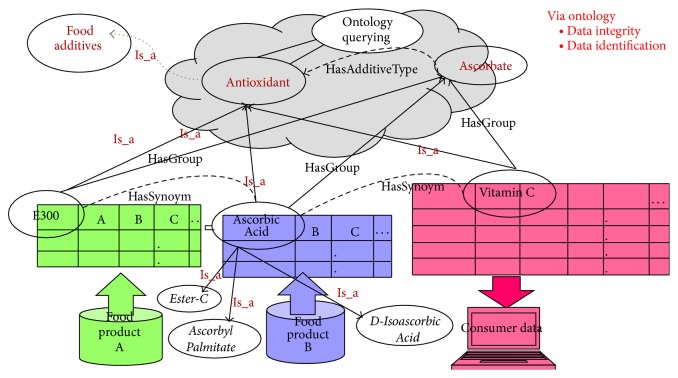
Semantic enhancement of the concept matching engine (CME).

Assume that the food domain specifies the term “Ascorbic Acid” as having two synonyms: “Vitamin C” or its E-codex standard name “E300” (Figure 3). In addition, “Ascorbic Acid” is marked with “hasGroup name” as “Ascorbate” and “Is_a relation” of “Antioxidant,” which is connected to another “Is_a” relation of “Food Additive” (Figure 3) (http://www.ingredientswizard.com/e-numbers-overview/320-e300e399-antioxidants-acidity-regulators-). The meaning of this then is that “Ascorbic Acid” is a “Food Additive” and also an “Antioxidant” that has two synonyms, “Vitamin C” and “E300,” and is in the “Ascorbate” group within the food domain. In addition, also assume that the concept of side effects from food nutrients related to a consumer's health problem is “Ascorbic Acid” or “E300,” which appears on a food product's label. However, the consumer knows only that vitamin C has a side effect on him or her and does not know the meaning of “Ascorbic Acid” or “E300.” A smart system needs to recognize the risk, define an intolerance score, and warn the consumer before he or she consumes the selected food product. Thus, the system considers the entire set of related concepts, properties, individuals, or synonyms according to the* consumer intolerance list*.

As a result, if the consumer chooses a packaged product on a market shelf during shopping, assume that it involves an E-codex standard name such as “E300.” Then, the CME of the system is able to recognize relational concepts such as synonym, “Is_a,” or “hasGroup” associated with “E300.” These related concepts are retrieved from the FOKB and then are saved in the* semantically enhanced consumer intolerance list* to use in the concept-matching step (second step) of the CME. Therefore, the system can determine that “Ascorbic Acid,” “E300,” “L-Ascorbic Acid,” “Ascorbate,” or “Vitamin C” are not healthy nutrients for that consumer and, for example, may cause some skin allergy problems for her or him. Then, the relational concepts are collected in another list as a new list,* semantically enhanced consumer intolerance list*, with entries such as “Ascorbic Acid,” “E300,” “L-Ascorbic Acid,” “Ascorbate,” or “Vitamin C.”

As a next step, the system starts to run its second step (concept matching) by considering the two lists below:

- *semantically enhanced consumer intolerance list (C):* all concepts of the nutrients, food additives, food compounds, and food items of which the consumer may be intolerant according to his or her health conditions,
- *selected product ingredients list (P):* concepts of the nutrients, food additives, food compounds, and food items of the selected food product.

The advantage of a semantic-based search is that the search domain is understood by machines. Given the idea “what am I looking for?” and the possible answer “I am looking for information about Ascorbic Acid,” when searching an ontology knowledge base for “what is Ascorbic Acid?” the system defines it as “Ascorbic Acid is an Ascorbate Food Additive and also an Antioxidant,” used as an “Acidity Regulator” and known as Vitamin C. As a result, a concept-matching algorithm can distinguish between search domains by using ontologies. If we use only syntactic-based search mechanisms in such systems, then we may end up with risky and harmful nutrition.

### 3.2. Mathematical Perspective for the Concept Matching Step of the Concept Matching Engine (CME)

The inputs of the CME are the concepts of the involved ingredients of the* selected product ingredients list*, *P*, and the* semantically enhanced consumer intolerance list*, *C*. The output is a set of matching or not matching nutrients with an intolerance score for the product according to the consumer intolerance list. The CME focuses on the concepts in these two lists of nutrients, food additives, food compounds, food items, and so forth, which are symbolized as *P* and *C* above. The CME assigns some predefined matching scores according to existing relations of the concepts in these two lists, where Dissimilar = 0, Subsume = 0.5, Plugin = 0.75, and Exact = 1. The four degrees of similarity are related as follows: Dissimilar < Subsume < Plugin < Exact [11]:

 
*C*: the set of concepts of the* semantically enhanced consumer intolerance list*,
 
*P*: the set of concepts of the* selected product ingredients list*,
 
*D*: the set of all concepts/classes defined in the food ingredients/compounds of the FOKB.

*Exact Relation*. A 1-1 mapping *C* → *P* where ∀*x*_*C*_*i*__ ∈ *C* and ∃*x*_*P*_*j*__ ∈ *P* such that Concepts(*x*_*C*_*i*__) ≡ Concepts(*x*_*P*_*j*__): any two focused concepts (*C*_*i*_ and *P*_*j*_) of both lists with the same or equivalent concepts or having a hasSynonym( ) relation.

 For instance, if (*C*_*i*_ = “Ascorbic Acid” and *P*_*j*_ = “E300”) or (*C*_*i*_ = “E300” and *P*_*j*_ = “Vitamin C”) or (*C*_*i*_ = “Vitamin C” and *P*_*j*_ = “E300”) or (*C*_*i*_ = “Vitamin C” and *P*_*j*_ = “L-Ascorbic Acid”), then an exact relation exists, giving a score of 1.

*Plugin Relation*. A 1-1 mapping from *C* → *P* where ∀*x*_*C*_*i*__ ∈ *C* and ∃*x*_*P*_*j*__ ∈ *P* such that Concepts(*x*_*C*_*i*__) ⊂ Concepts(*x*_*P*_*j*__): if any focused concept (*C*_*i*_) of the* semantically enhanced consumer intolerance list (C)* is a subset or subclass of the concept of* selected product ingredients list (P)*'s input (*P*_*i*_) or having a hasGroup( ), hasAdditiveType( ), or hasIs_a( ) relationships, then a Plugin relationship exists.

 For instance, if (*C*_*i*_ = “Vitamin C” and *P*_*j*_ = “Ascorbate”) or (*C*_*i*_ = “E300” and *P*_*j*_ = “Ascorbate”) or (*C*_*i*_ = “Ascorbic Acid” and *P*_*j*_ = “Ascorbate”) or (*C*_*i*_ = “L-Ascorbic Acid” and *P*_*j*_ = “Ascorbate”), then a Plugin relation exists, giving a score of 0.75.

*Subsume Relation*. A 1-1 mapping from *C* → *P* where ∀*x*_*C*_*i*__ ∈ *C* and ∃*x*_*P*_*j*__ ∈ *P* such that Concepts(*x*_*C*_*i*__)⊃Concepts(*x*_*P*_*j*__): if any focused concept (*C*_*i*_) of the* semantically enhanced consumer intolerance list (C)* is a superclass of the concept of the* selected product ingredients list (P)*'s input (*P*_*j*_) or has a hasGroup( ), hasAdditiveType( ), or hasIs_a( ) relationship, then a Subsume relationship exists.

 For instance, if (*C*_*i*_ = “Ascorbate” and *P*_*j*_ = “Vitamin C”) or (*C*_*i*_ = “Ascorbate” and *P*_*j*_ = “E300”) or (*C*_*i*_ = “Ascorbate” and *P*_*j*_ = “Ascorbic Acid”) or (*C*_*i*_ = “Ascorbate” and *P*_*j*_ = “L-Ascorbic Acid”), then a Subsume relation exists, with a score of 0.5.

*Dissimilar Relation*. A 1-1 mapping from *C* → *P* where ∀*x*_*C*_*i*__ ∈ *C* and ∃*x*_*P*_*j*__ ∈ *P* and Concepts(*x*_*C*_*i*__) ≠ Concepts(*x*_*P*_*j*__): if there is no relation between the inputs of the two lists, a Dissimilar relationship exists.

 If (*P*_*j*_ = “Vitamin C” and *C*_*i*_ = “Phytic acid”), then a Dissimilar relation exists, which is scored as 0. Clearly, the following relations hold among the concepts of these lists *P*⊆*D*, *C*⊆*D*. The similarity score is computed using Algorithm 1.

Calculating the degree of similarity matching of the parameters of on-focus two concepts in the **C** and **P** lists is defined in Algorithm 1. If the degree of matching is high, then the intolerance effect is high for the consumer according to Algorithm 1. The Exact relation indicates that the consumer has high intolerance of the considered food additive (***Concept*** **P**_**j**_) in the selected product.

As shown in Algorithm 2,  ***getIntoleranceScore***(**C** ***List***,  **P** ***List***) uses two lists, **C** and **P**, to find the total similarity score in the second step of the CME. The first parameter is a list of concepts of the ingredient side effects concepts **C** = {**C**_1_, **C**_2_,…, **C**_**m**_}, while the second is a list of concepts of selected product's ingredients **P** = {**P**_1_, **P**_2_,…, **P**_**n**_}. During this step, the* hasGroup( )*,* hasAdditiveType( )*,* hasSynonym( )*, or* hasIs_a( )* properties or class IRI concepts of the* semantically enhanced consumer intolerance list (C)* are analyzed by recovering their semantics, that is, meaning, similarities, differences, and relations, while carrying out Algorithm 1. Similarly, the semantic distances between concepts, which offer similarity information between concepts, can be provided by the ontology developer during the development phase:

(1) $$\begin{array}{c} d_{weightA}=\frac{1}{\#ofSubconceptsofA}. \end{array}$$

If semantic distances are not scored by the developer, all direct subconcepts of a parent concept will have the same distance weight (Bener et al. [12–14]) according to (1).

The CME finds the semantic distance weight *d*_*weight*(*A*,*Z*)_ between any two concepts **A** and **Z** in a particular domain ontology as given by (2):

(2) dweightA,Z=dweightA,B∗dweightB,C∗⋯∗dweightW,Y∗dweightY,Z.

The applied CME scoring method is a simple multiplicative weighting function. A given ingredient side effects concept list *C* with input concepts **C** = {**C**_1_, **C**_2_,…, **C**_**m**_} and a product **P** with ingredient concepts **P** = {**P**_1_, **P**_2_,…, **P**_**n**_} are matched, and the total score *IntoleranceScore*(*C*, *P*) is calculated according to (3) (Çelik and Elçi [15]):
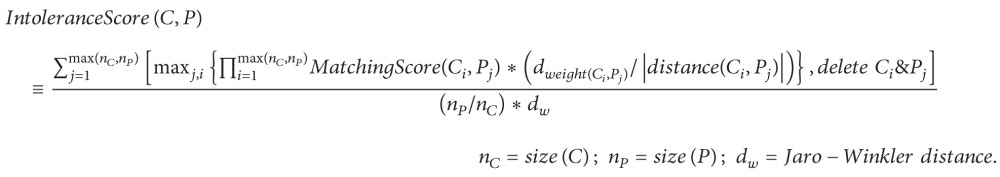
(3)Before going into details of the usage of the formulas given above in the proposed system, some formal information should be provided about these subconcepts since they are commonly used as food additives, as presented in the literature discussed below. According to the Micronutrient Research for Optimum Health of the Linus Pauling Institute (http://lpi.oregonstate.edu/infocenter/vitamins/vitaminC/vitCform.html), Ester C contains mainly Calcium Ascorbate but also contains small amounts of the Vitamin C metabolites, Dehydroascorbic Acid (oxidized Ascorbic Acid), Calcium Threonate, and trace levels of Xylonate and Lyxonate. Ester C should not be confused with Ascorbyl Palmitate, which is also marketed as “Vitamin C Ester”. The roles of Vitamin C in promoting collagen synthesis and as an antioxidant have generated interest in its use on the skin (http://lpi.oregonstate.edu/infocenter/skin/vitaminC/index.html). Ascorbyl Palmitate is a fat-soluble antioxidant used to increase the shelf life of vegetable oils and potato chips [16]. Ascorbyl Palmitate is frequently used in topical preparations because it is more stable than some aqueous (water-soluble) forms of Vitamin C [17]. Ascorbyl Palmitate is also marketed as “Vitamin C Ester,” which should not be confused with Ester C (as mentioned above). The last type of Ascorbic Acid is D-Isoascorbic Acid (Erythorbic Acid). Erythorbic Acid is an isomer of Ascorbic Acid. Erythorbic Acid is used in the US as an antioxidant food additive and is generally recognized as safe. A series of studies in young women found that up to 1,000 mg/day of Erythorbic Acid for as long as 40 days was rapidly cleared from the body and had little effect on the bioavailability of Ascorbic Acid, indicating that Erythorbic Acid does not diminish the bioavailability of Ascorbic Acid in humans at nutritionally relevant levels of intake [18].

The above equation considers* two lists* **C** = {**C**_1_, **C**_2_,…, **C**_**m**_} and **P** = {**P**_1_, **P**_2_,…, **P**_**n**_} of the **C** and **P**. In (3),* distance*(**C**_**i**_, **P**_**j**_) shows the number of concepts (levels) between any two focused concepts, **C**_**i**_ and **P**_**j**_, in the ontology. Assume that the ***Ascorbic Acid*** concept has three subconcepts in the ontology, such as ***Ester C***,** Ascorbyl Palmitate (Vitamin C Ester)**, and** D-Isoascorbic Acid (Erythorbic acid)** (Figure 5). Also assume that the* semantically enhanced consumer intolerance list (C)* contains the** D-Isoascorbic Acid (Erythorbic Acid)** concept since the consumer may have some skin problems when consuming it. In addition, assume that the consumer may choose a product on the market shelves that contains** Ascorbic Acid**. Therefore, the value of the *d*_*weight*(*AscorbicAcid*, *Dehydroascorbic* *Acid*)_ will be 1/3, that is, rounded as 0.333.

Therefore, the **C**_**i**_ =** Dehydroascorbic Acid** *i*th input concept of** C** and the **P**_**j**_ =** Ascorbic Acid** *j*th input concept of** P** are considered; then the *MatchingScore*(*C*_*i*_, *P*_*j*_)*∗*(*d*_*weight*(*C*_*i*_,*P*_*j*_)_/|*distance*(*C*_*i*_, *P*_*j*_)|) of the (**Dehydroascorbic Acid, Ascorbic Acid**) match is (0.75*∗*0.333)/1 = 0.250. Because (**Dehydroascorbic Acid, Ascorbic Acid**) has a** PLUGIN** relation, the subsumption score is 0.75 according to Algorithm 1, and the weight is 0.333 by (1).

Moreover, there exists a subclass relation between (**Dehydroascorbic Acid, Ascorbic Acid**) and thus {**d** **i** **s** **t** **a** **n** **c** **e** (**Dehydroascorbic Acid**, **A** **s** **c** **o** **r** **b** **i** **c** **A** **c** **i** **d**)} is 1. The concepts used above are depicted in the** FOKB** through “**Is_a**” relations (Figure 3).

**Figure 4:**
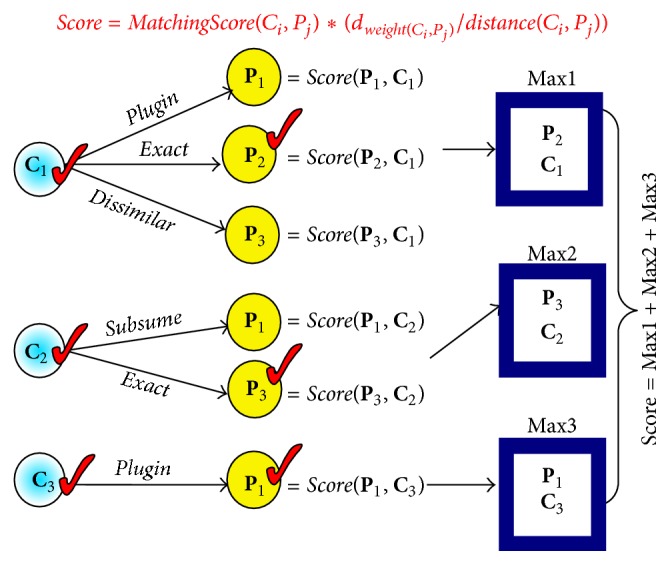
A simple example using Algorithm 2.

**Figure 5:**
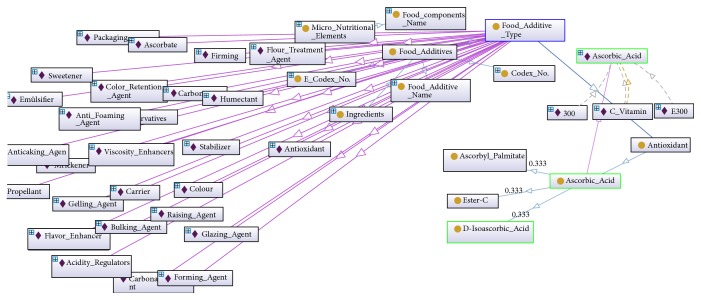
**Ester C**,** Ascorbyl Palmitate** (**Vitamin C Ester**), and** D-Isoascorbic Acid** (**Erythorbic Acid**) are three subconcepts of the** Ascorbic Acid** concept, and each link has 0.333 weight value.

As shown in Figure 4, the system takes as input the focused ingredient side effects concepts** C** for a consumer and iterates over every** p** in the** P** list to determine a match. The** C** and** P** lists match if their concepts each are matched according to one of the* Dissimilar, Subsume, Plugin,* or* Exact* relationships. Figure 4 illustrates the basic step in the***getIntoleranceScore***(**C*List***,** P*List***) function by means of a simple example. The solid lines indicate the relationships inferred by the CME.

For example, the input sets of **C** = {**C**_1_, **C**_2_, **C**_3_} and **P** = {**P**_1_, **P**_2_, **P**_3_} are executed by the ***getIntoleranceScore***(**C*List***,** P*List***) in the CME. Pseudo code for the ***getIntoleranceScore***  function of Algorithm 2 is given above. The algorithm first attempts to compute a maximum match for **C**_1_ (as depicted in Figure 4). Assume that the following matches are inferred, then the values of *Score* = *MatchingScore*(*C*_*i*_, *P*_*j*_)*∗*(*d*_*weight*(*C*_*i*_,*P*_*j*_)_/|*distance*(*C*_*i*_, *P*_*j*_)|) are calculated by dividing each parameter of** C** by the** distance**(**C**_**i**_, **P**_**j**_) value:

 
**C**_1_ *subsumes* **P**_1_ → **P** **l** **u** **g** **i** **n**,
 
**C**_1_ *is a synonym* of **P**_2_ → **E** **x** **a** **c** **t**,
 
**C**_1_ does* not match* **P**_3_ → **D** **i** **s** **s** **i** **m** **i** **l** **a** **r**.

**C**_1_ has two relations that are one** Exact** and one** Plugin**. However, the CME takes a max match with **P**_2_ that is Exact. Thus, **P**_2_ and **C**_1_ are removed from their respective lists. The algorithm then attempts to match the next concept, **C**_2_. Assume that the following matches are inferred:

 
**C**_2_ *subsumes* **P**_1_ → **S** **u** **b** **s** **u** **m** **e**,
 
**C**_2_ *is a synonym* of **P**_3_ → **E** **x** **a** **c** **t**.

Thus, **C**_2_ is matched with **P**_3_; therefore, **P**_3_ and **C**_2_ are removed from their respective lists. The algorithm then attempts to find a match for **C**_3_. Assume that the following match is inferred for the last parameter in **C**:

 
**C**_3_ *subsumes* **P**_1_ → **P** **l** **u** **g** **i** **n**.

If the sizes of the **C** and **P** are not equal, we insert |**s** **i** **z** **e**(**C**) − **s** **i** **z** **e**(**P**)|* null* values into the smaller list. Thus, the function calculates the maximum values for each iteration and then finds their sum.

Note that (3) also takes into account the overall distance using the* Jaro-Winkler* distance [19, 20] and also the ratio of concept sizes of the **C** and **P** lists to complete the calculation of the average *IntoleranceScore*(*C*, *P*) for the given (**C**, **P**) query. The same equations and operations are also applied to the next products to define *IntoleranceScore*(*C*, *P*) of each chosen product according to the intolerance list of the consumer.

One of the key differences between syntactic/lexical and semantic matching is that in syntactic matching, when two concepts are matched, we consider only the labels attached to them, independent of the position of the concepts in the ontology graph. On the other hand, when matching two concepts in semantic matching, the concepts we analyze depend not only on the concept attached to the node, but also on the position of the concept in the ontology. Furthermore, we also consider the Jaro-Winkler distance, which was designed and is best suited to match strings to measure the syntactic similarity between two terms. The Jaro-Winkler distance eliminates duplicate records in database tables and also normalizes the score, such that 0 denotes dissimilarity and 1 is an exact match. Therefore, the Jaro distance **d**_**j**_ is modified for use with the lists of concepts of the **C** and **P** instead of being applied to two given strings. For semisemantic-based input matching among concepts, the formula based on the* Jaro-Winkler distance* approach is modified as given below:

(4) dj=12∗mnC+mnP,nC=sizeC;  nP=sizeP;

- **m** is a value based on the number of matching concepts multiplied by their particular degrees, for any theoretic relation including {≡, ∩, ⊂, ⊃}, but excluding Dissimilar {≠}, where *m* = (#ofExact*∗*1) + (#ofPlugin*∗*0.75) + (#ofSubsume*∗*0.5);
- **l** gives the length of the found relations of all the concepts;
- **k** is the constant scaling factor denoting how much the score has been adjusted upwards for common concepts; the standard value for this constant in Winkler's work is **k** = 0.1;
- the Winkler distance **d**_**w**_ is *d*_*w*_ = *d*_*j*_ + (*l*.*k*.(1 − *d*_*j*_)),

where **d**_**j**_ is the Jaro distance for all the concepts of **C** and **P**.

Note that (4) always yields a value between 0 and 1, since the total number of correspondences in **m** cannot be greater than the average size of the two sets **C** and **P**. Assume three given input lists, a client intolerance list (**C**), and ingredients of two products (**P**_1_ and **P**_2_), as listed in the following (Table 11).

*(i) Define Synonym (Exact) Relations*. The** Ascorbic Acid** concept has three synonyms:** E300**,** Vitamin C**, and** L-Ascorbic Acid**. In addition, the** Vitamin** **B**_3_ concept has two synonyms:** Niacin** and** Niacinamide** (see Table 1). Finally, the** Algin** concept has one synonym:** Alginic acid**.

(a) Therefore, for Vitamin **B**_3_ and Niacin, consider
  (5) MatchingScoreVitamin  B3,Niacin=1ExactdweightVitamin  B3,Niacin=1synonymddistanceD-Isoascorbic  Acid,Ascorbic  Acid=1synonym.

*(ii) Define Sub- or Super Class (Plugin or Subsume) Relations*. The** Ascorbic Acid** concept has three direct subconcepts in the ontology:** Ester-C**,** Ascorbyl Palmitate** (**Vitamin C Ester**), and** D-Isoascorbic Acid** (**Erythorbic Acid**).

(a) Therefore, for D-Isoascorbic Acid and Ascorbic Acid, consider
  (6) MatchingScoreD-Isoascorbic  Acid,Ascorbic  Acid=0.50SubsumedweightD-Isoascorbic  Acid,Ascorbic  Acid=13=0.333three  direct  subclassesddistanceD-Isoascorbic  Acid,Ascorbic  Acid=1direct  link.
(b) Therefore, for D-Isoascorbic Acid and Ascorbyl Palmitate, consider
  (7) MatchingScoreD-Isoascorbic_Acid,Ascorbyl_Palmitate=0.50  SubsumedweightD-Isoascorbic_Acid,Ascorbyl_Palmitate=0.333∗0.333weights  of  the  two  links  between  themddistanceD-Isoascorbic_Acid,Ascorbyl_Palmitate=2indirect  links.

In addition, the** Algin** concept has two direct subconcepts in the ontology:** Calcium Alginate** and** Sodium Alginate**.

(a) Therefore, for Algin and Sodium Alginate, consider
  (8) MatchingScoreAlgin,Sodium_Alginate=0.75PlugindweightAlgin,Sodium_Alginate=12subclasses=0.50ddistanceAlgin,Sodium_Alginate=1direct  link.
(b) Therefore, for Algin and Alginic Acid, consider
  (9) MatchingScoreAlgin,Alginic_Acid=1ExactdweightAlgin,Alginic_Acid=1synonymddistanceAlgin,Alginic_Acid=1synonym.

*(iii) Define Jaro-Winkler for* **P**_1_ *and* **P**_2_ *Products*

(a) For **P**_1_ ingredients, *m* = 2 since there are 2 matched concepts:
  (10) D-Isoascorbic  Acid  Subsumed  by  Ascorbic  Acid⟶SubsumeAlgin  Subsumes  Sodium  Alginate⟶PluginnC=3,nP=3.

We calculate a Jaro score of

(11) dj=12∗mnC+mnP=121∗0+1∗0.50+1∗0.753+1∗0+1∗0.50+1∗0.753=0,4167.

The Jaro-Winkler scores using the standard weight **k** = 0.1 and **l** = 3 same as **m** are given by

(12) dw=dj+l.k.1−dj=0,4167+2∗0.1∗1−0,4167=0,53336IntoleranceScoreC,P1≡0∗0+0.50∗0.333/1+0.75∗0.5/13/3∗0.53336=0,1665+0,3751,60008=1,015IntoleranceScoreC,P2=1.015∗1003max  score  for  #  of  matched  concepts=%33  have  negative  effects.

(b) For **P**_2_ ingredients, *m* = 3 since there are 3 matching concepts:
  (13) D-Isoascorbic  Acid  Subsumed  by  Ascorbyl  Palmitate⟶SubsumeVitamin  B3  Synonym  Niacin⟶ExactAlgin  Subsumes  Sodium  Alginate⟶PluginnC=3,nP=3.

We calculate a Jaro score of

(14) dj=12∗mnC+mnP=121∗0.50+1∗1+1∗0.753+1∗0.50+1∗1+1∗0.753=0.75.

The Jaro-Winkler scores using the standard weight **k** = 0.1 and **l** = 3 same as **m** are given by

(15) dw=dj+l.k.1−dj=0,75+3∗0.1∗1−0,75=0,825IntoleranceScoreC,P2≡0.50∗0.333∗0.333/2+1∗1/1+0.75∗0.5/13/3∗0.825=0.0277+1+0.3750.825=1,7IntoleranceScoreC,P2=1.7∗1003max  ⁡score  for  #  of  matched  concepts=%56  have  negative  effects.

Note that (3) considers the overall distance via the** Jaro**-**Winkler** distance (**d**_**w**_) by multiplying by the ratio of the concept sizes of the **C** and **P** lists to compute the final average *IntoleranceScore*(*C*, *P*) for the given (**C**, **P**) query.

Finally, intolerance score values for Product 1 and Product 2 are 33% and 56%, respectively. The high intolerance score of 56% for Product 2 gives the idea to the consumer that Product 2 is nearly 56% harmful since it involves more ingredient side effects than Product 1.

**Figure 6:**
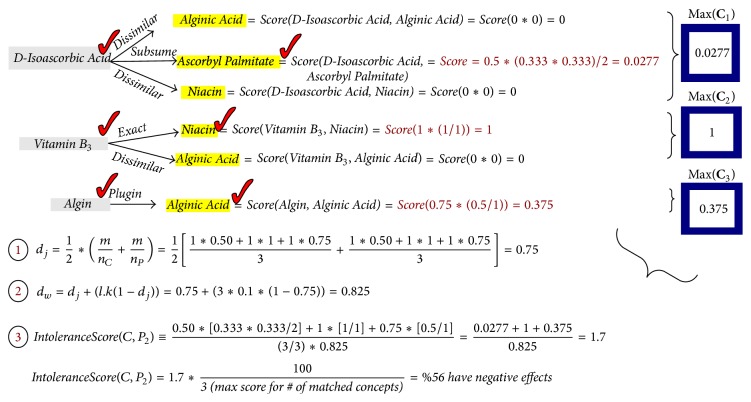
CME calculations of intolerance score for matching harm potential and** Product 2**.

Figure 6 presents the steps for calculation of the intolerance score by matching** Consumer** and** Product 2** (by using the equations in the figure).** Product 2** is found to be more harmful for the** Consumer** since it has the higher intolerance score after concept matching.

Matching of the** Consumer** and** Product 2** lists gives the highest score according to concept matching since there are one** Subsume**, one** Exact**, and one** Plugin** relation. All applied calculations of the CME are shown in Figure 6.

## 4. Inferencing via Semantic Web Rule Language (SWRL)

Beyond the abovementioned contributions of the FOKB, such semantic declarations are also used to infer meaningful and relevant information from the preasserted data of food products. That is done through an inference mechanism which is able to make an appropriate suggestion of a food given a specific health condition of a consumer. In addition, Semantic Web Rule Language (SWRL) is an expressive OWL-based rule language that provides more powerful deductive reasoning capabilities than OWL alone. The SWRL is built on the same descriptive logic foundation as OWL and provides similar strong formal guarantees when performing inference tasks. A SWRL rule contains an antecedent (body) and a consequence (head). Both the body and head consist of positive conjunctions of atoms: *BODY*{*Atom*∧*Atom*…} → *HEAD*{*Atom*∧*Atom*…}. An atom is an expression of the form that contains a predicate symbol, such as *P*, and also some parameters, such as *par*_1_, *par*_2_,…, *par*_*n*_. The predicate symbol *P* can represent OWL classes, object properties, or data type properties. *P* may contain some parameters that can be OWL individuals or data values or variables referring to them in the expression: *P*  (*par*_1_, *par*_2_,…, *par*_*n*_), such as *hasAge*(?*p*, ?*age*)*or* *greaterThan*(?*age*, 18), where “?**p**” is a variable parameter used instead of an individual of the “**Person**” class. The term “?**a** **g** **e**” is a variable parameter that is used to hold the age information of a person. The **g** **r** **e** **a** **t** **e** **r** **T** **h** **a** **n** predicate takes also two parameters: the first parameter “?**a** **g** **e**” holds a value that is “18” as the second parameter in the example given above.

The allergy rules are semantically defined by using SWRL for both the English and Turkish languages. The SWRL rule declarations are applied to predefined criteria to find allergies of a person according to the food additives, food items, e-codex, and so forth associated with the chosen product. Therefore, the rule declarations are modeled in the form of SWRL, and the system uses its own predefined rules to find allergen items in the selected food product on the market shelves.

For instance,

 
**Consumer**(**?c**),** Has_Allergy(?c**,** Lactose_Allergy**),** Has_Ean_No**(**?c**,** ?p**),** Has_Product_Additives_Name**(**?p**,** Aluminium_Silicate**) ->** HAS_LACTOSE_RISK**(**?c**,** Aluminium_Silicate**)

The meaning of the above rule is as follows:

- if the consumer “**c**” has an allergy,
- also if the allergy is “Lactose_Allergy,”
- also if the consumer “**c**” chooses the product “**p**,”
- also if the product “**p**” involves “Aluminium_Silicate” then (→),
- the consumer “**c**” has HAS_LACTOSE_RISK as a new property assigned because of the “Aluminium_Silicate” food additive.

“Aluminium_Silicate” has synonym (exact) relations, such as Kaolin, and also has plugin/subsume relations, such as E554: Sodium Aluminium Silicate, E555: Potassium Aluminium Silicate, and E556: Calcium Aluminium Silicate (http://www.codexalimentarius.net/gsfaonline/additives/details.html?id=294). Therefore, the matching task of CME will be able to define if the product involves one of these concepts (Kaolin, E554, E555, E556, Sodium Aluminium Silicate, Potassium Aluminium Silicate, and Calcium Aluminium Silicate) that have a relation (such as Exact, Plugin, or Subsume) with the allergen “Aluminium_Silicate” concept of the person when running the rule beforehand.

**Figure 7:**
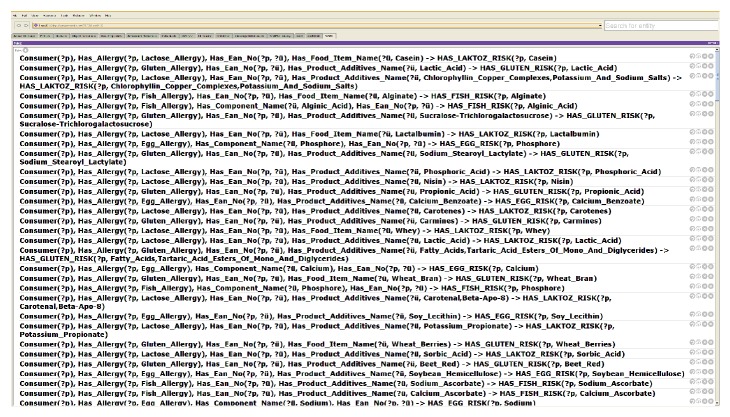
SWRL rule set of the system.

The current FOKB contains four main classes, 58 subclasses, 15 object type properties and 17 subobject type properties, 12 data type properties, 1530 individuals with annotation type properties (Figure 1), and 210 semantic rules (Figure 7 or Box 1). Some of the rules are presented in Box 1. The rules consider four types of risks through OWL object properties: HAS_LACTOSE_RISK, HAS_GLUTEN_RISK, HAS_FISH_RISK, and HAS_EGG_RISK. Some of the negative effects of the ingredients gluten, lactose, egg, and fish are presented in Box 1. A gluten allergy for example will be discussed as a case study in the next section.

## 5. Case Study

People continue to consume food products without knowing what is in them when they are purchased from market shelves. The aim of this study was to identify and match food additives on food packaging in order to observe sensitivity of consumers. Therefore, this section discusses a case study of a consumer striving to select food safe for the consumer's own consumption while shopping for food products on the shelves of a big market. For this purpose, this consumer uses the proposed system's mobile application. The customer could also use the system via kiosks (in the big markets) or any smart device such as phones and iPad. After the login task, the system will need to scan the QR code/barcode of a food product that is tagged on the product packaging. The QR code contains an EAN number of the food product. Then, the system will get all the ingredient lists and energy details of the scanned product via its EAN number from database. Querying the database, the system connects to the food search web services of the Republic of Turkey Ministry of Food Agriculture and Livestock (http://www.tarim.gov.tr/Sayfalar/Eng-1033/Anasayfa.aspx#).

The system starts to search for side effects of food additives and energy details according to the consumer's health condition (food allergy, heart disease, hypertension, cholesterol, diabetes, etc.) through the SWRL rule set of the FOKB. After that, the system will return any intolerance reasons found through the SWRL rules and the score of the product after CME matching according to the food nutrients sensitivity of the customer.

**Figure 8:**
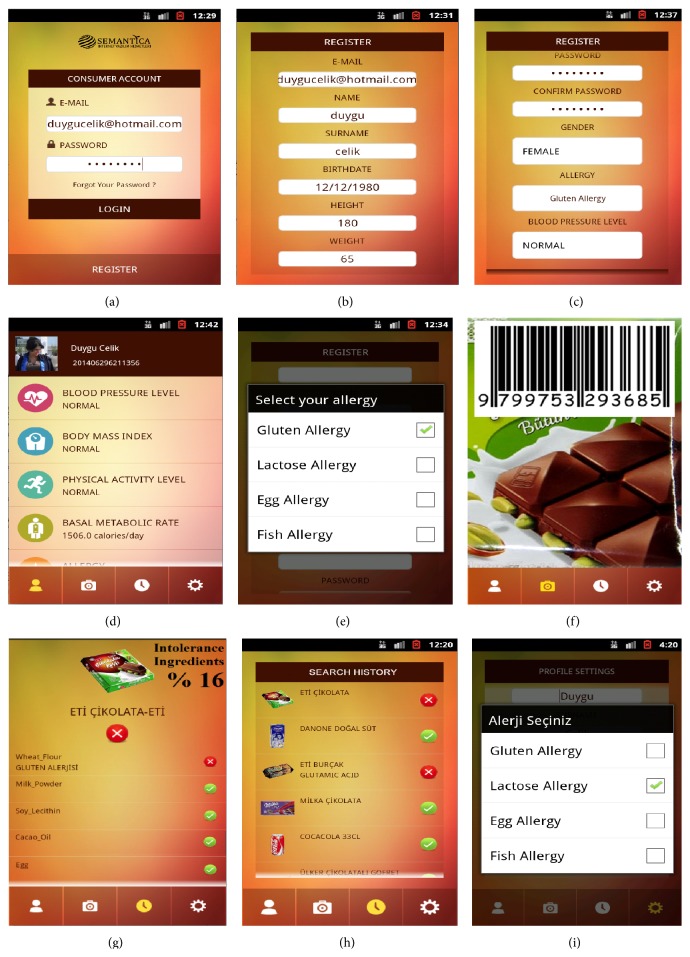
The customer member chooses various products and gets the results on the android mobile application of the system though FOKB.

In our scenario, Ms. Celik, who has gluten allergy, logs into the application system while she is shopping in a supermarket. She has a smart phone that is able to connect to the FoodWiki mobile application. She connects to the system via her username and password (Figure 8(a)). If the consumer has not registered before, then she can create a new user account for herself (Figures 8(b) and 8(c)). She can see the profile details as is shown in Figure 8(d). She wants to check a product on the market shelves in order to buy suitable ingredients for her health condition, that is, gluten allergy (Figure 8(e)). She then chooses a packaged food product from a shelf (e.g., the product is** ETİ** chocolate in Figure 8(f)). She wants to be sure that the chosen product will not cause any side effects for her condition since she has intolerances for the gluten risk type of wheat, glutamic acid, and wheat flour, and so forth. She scans the QR code/barcode of the product on her mobile phone (or alternatively through a market kiosk) and then the system retrieves all the nutritional information related to that product from the database of the ministry through its search product web services (Figure 8(f)). Then, the system searches the nutritional information to get the indicated concepts in the FOKB. In addition to retrieving the nutritional concepts of commercial products, the system is able to compare them with specific nutritional concepts affecting her health (i.e., see the warning messages and intolerance rate on screens; Figure 8(g)).

The system presents three different colors as a result to the consumer: red, green, and yellow. The green light indicates “the product is safe” for the consumer while the red light is an “objectionable food product” (the red is indicated if the CME finds at least one common ingredient that is defined as having an exact relationship between the consumer intolerance list and product ingredient list; Figure 8(h)). In addition, the yellow light indicates a possible issue, so the consumer should seek professional medical advice before consuming that product (the yellow is considered if the CME does not find any exact relationship between the consumer intolerance list and the product ingredient list that involves side effects from ingredients).

If the consumer wants to update her profile, then she can do it via the update window (Figure 8(i)). Figure 8(h) shows that the customer has tried various different products and has gotten some suitable product lights (result is green) or some unsuitable product lights (result is red) in the history window of the application. The system shows a result message explaining reasons for the suitability of the product for the customer (i.e., the system explains why the result is green, yellow, or red). In addition, the system shows general information about each ingredient in the product list, such as amount of nutrients, additives/compounds information, energy information, and the recommended daily intake.

## 6. Tools Employed

In developing the FOKB, Protégé 4.3 [9] with OWL 2.0 support is the preferred tool. Java (http://java.sun.com/products/archive/j2se/6u7/index.html) programming language is used in the functional architecture of the system; parsing over the FOKB is through Java-based Ontology Parser OWL API ver 3.4.10 [21]. The Pellet [22] OWL reasoner supports reasoning with SWRL rules. Pellet interprets SWRL using the DL-Safe Rules notion, which means rules will be applied only to the named individuals in the ontology. The SWRL rules are applied to infer the colored results to identify appropriate food products for individuals (consumers). In addition, the Pellet API (http://clarkparsia.com/pellet/) for Java is used in programming to generate inferences via predefined SWRL rules of the FOKB in the Java environment.

Important algorithms of the methodology base of the system and also some inspired present semantic matchmaking studies are discussed in Section 3. Based on these methods, the concepts related to a consumer's sensitive food nutrients information are matched with the concepts of the scanned or queried food product through human, disease, and food ontologies (semantic matching), and then the returned result is provided as a suggested consuming decision to the consumer. It identifies potential side effects to the consumer as a report on the product's appropriateness before a consuming decision is made. The flow diagram of the implemented mobile application of the system is shown in Appendix B on Java that runs an inferencing mechanism through the SWRL rule set of the FOKB to infer side effects in a product chosen by a consumer according to his or her intolerance list.

## 7. Conclusion

The paper describes briefly the design steps, working mechanism, and case use of the proposed Ontology-Driven Mobile Safe Food Consumption System (FoodWiki) using semantic matching. The system is especially designed for examining packaged food products on market shelves and suggesting the selected product's appropriateness to food consumers according to their health conditions or intolerances. The system uses its own ontology knowledge base that involves four subsections: person, disease, product, and food ingredients/compounds. The ingredients/compounds involve nutritional information about commercial products from the food field and chemical perspective. This system ontology knowledge base is used to share knowledge among mobile smart devices of food consumers and the product database of the Republic of Turkey Ministry of Food, Agriculture and Livestock via ontology parser web services. Currently, we address only the design of the food consumption suggestion system in terms of provision of nutritional advice to market consumers according to their food intolerances or health conditions.

The system makes use of its knowledge base through its* concept matching engine* (*CME*) that performs two sequenced tasks:* semantic enhancement of concepts* as a first step and then a* concept matching step*. The CME obtains a total intolerance score that expresses the degree of intolerance for each chosen product according to individual consumer intolerance concepts. While calculating the score, each concept in the consumer intolerance collection is compared against the ingredient collection concepts of a chosen product on market shelves. All required details of the proposed semantic similarity-based intolerance scoring mechanism have been formulated and presented by giving algorithms used in the CME. Additionally, we presented a* food consuming* scenario that features* gluten allergy* and choice of a chocolate product on the market shelves and demonstrates use of the android mobile application of the system. The application is developed for various platforms such as mobile applications of IOS, Android, and Windows that are currently in the testing stage.

## Figures and Tables

**Figure 9:**
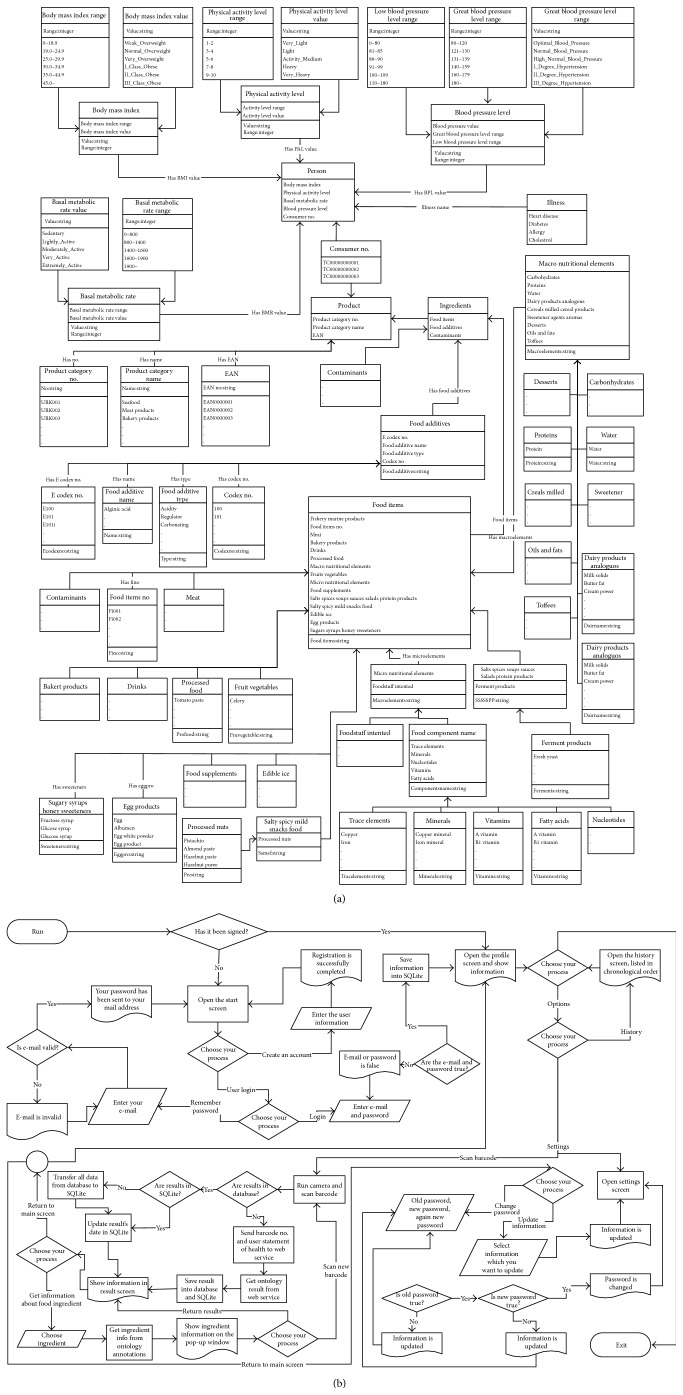
(a) Class diagram of FOKB. (b) Flow diagram of FoodWiki mobile system.

**Box 1:**
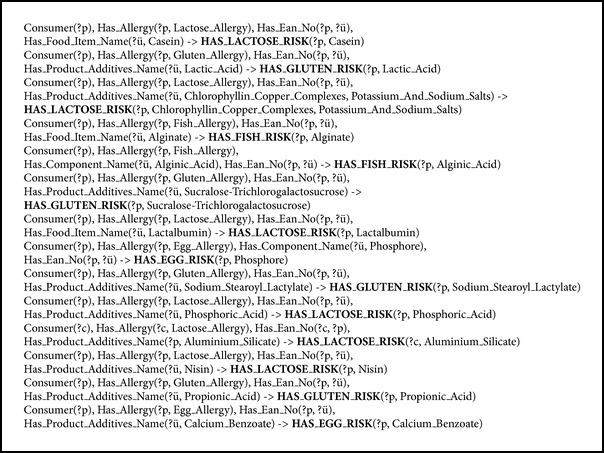
The defined SWRL rules for four-type allergies/intolerances, gluten, lactose, egg, and fish.

**Algorithm 1:**
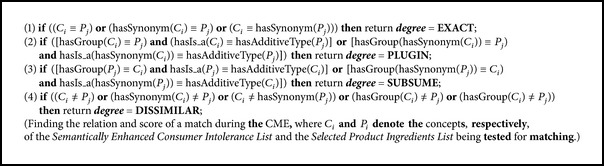
MatchingScore(Concept *C*_*i*_, Concept *P*_*j*_).

**Algorithm 2:**
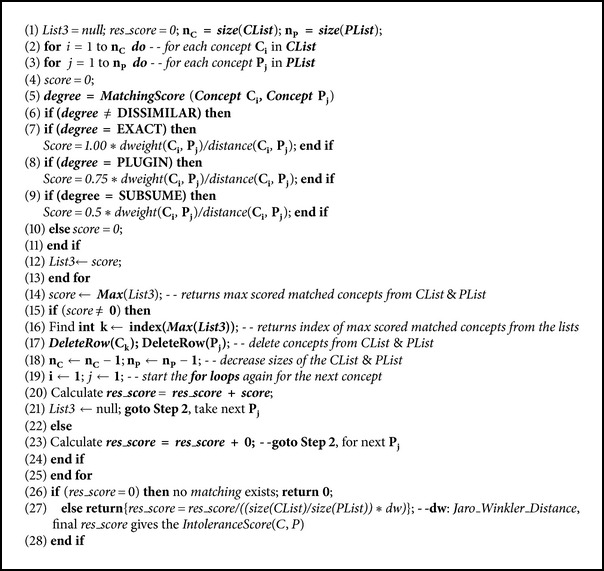
*getIntoleranceScore (CList, PList)*.

**Table 1:** Initial section of the food ontology knowledge base (FOKB).

Main classes	Subclasses
Thing → diseases → allergy	(i) Leguminous allergy, (ii) fish allergy, (iii) wheat protein allergy, (iv) nut seed allergy, (v) gluten allergy, (vi) lactose intolerance, (vii) fruits and vegetables allergy, (viii) milk protein allergy, (ix) egg allergy, (x) celiac disease allergy, and so forth

Thing → person	(i) Physical activity level, (ii) basal metabolic rate, (iii) blood pressure level, (iv) body mass index, (v) consumer, and so forth

Thing → ingredients	(i) Contaminants, (ii) food additives, (iii) food items, and so forth

Thing → product	(i) EAN, (ii) product category name, (iii) product category no., and so forth

**Table 2 tab2:** Physical activity level class includes two subclasses: physical activity level range and physical activity level value, with their values for individuals.

Main classes	Subclasses of the physical activity level	Values (individuals)	Relationships
Thing → person → physical activity level	Physical activity level range	(i) 1-2, (ii) 3-4, (iii) 5-6, (iv) 7-8, (v) 9-10	(i) 1-2 → very light, (ii) 3-4 → light, (iii) 5-6 → activity medium, (iv) 7-8 → heavy, (v) 9-10 → very heavy
	Physical activity level value	(i) Very light, (ii) light, (iii) activity medium, (iv) heavy, (v) very heavy	

**Table 3 tab3:** Basal metabolic rate class includes two subclasses: basal metabolic rate range and basal metabolic rate value, with their values for individuals.

Main classes	Subclasses of the basal metabolic rate	Values (individuals)	Relationships between the BMR range and value classes
Thing → person → basal metabolic rate	Basal metabolic rate range	(i) 0–800, (ii) 800–1400, (iii) 1400–1600, (iv) 1600–1900, (v) 1900–above	(i) 0–800 → sedentary, (ii) 800–1400 → lightly active, (iii) 1400–1600 → moderately active, (iv) 1600–1900 → very active, (v) 1900–above → extremely active
	Basal metabolic rate value	(i) Sedentary, (ii) lightly active, (iii) moderately active, (iv) very active, (v) extremely active	

**Table 4 tab4:** Blood pressure level class includes three subclasses: high blood pressure level range, low blood pressure level range, and blood pressure level value, with their values for individuals.

Main classes	Subclasses of the blood pressure level	Values (individuals)	Relationships between high and low BPL classes
Thing → person → blood pressure level	High blood pressure level range	(i) 80–120, (ii) 121–130, (iii) 131–139, (iv) 140–159, (v) 160–179, (vi) 180–above	(i) 0–80 & 80–120 → optimal blood pressure, (ii) 81–85 & 121–130 → normal blood pressure, (iii) 86–90 & 131–139 → high-normal blood pressure, (iv) 91–99 & 140–159 → degree I hypertension, (v) 100–109 & 160–179 → degree II hypertension, (vi) 110–180 & 180–above → degree III hypertension
	Low blood pressure level range	(i) 0–80, (ii) 81–85, (iii) 86–90, (iv) 91–99, (v) 100–109, (vi) 110–180,	
	Blood pressure level value	(i) Optimal blood pressure, (ii) normal blood pressure, (iii) high-normal blood pressure, (iv) degree I hypertension, (v) degree II hypertension, (vi) degree III hypertension	

**Table 5:** Body mass index class includes two subclasses: body mass index range and body mass index value, with their values for individuals.

Main classes	Subclasses of the body mass index	Values (individuals)	Relationships between the BMI range and value classes
Thing → person → body mass index	Body mass index range	(i) 19.0–24.9, (ii) 25.0–29.9, (iii) 30.0–34.9, (iv) 35.0–44.9, (v) 45.0–above	(i) 19.0–24.9 → normal overweight, (ii) 25.0–29.9 → very overweight, (iii) 30.0–34.9 → class I obese, (iv) 35.0–44.9 → class II obese, (v) 45.0–above → class III obese
	Body mass index value	(i) Normal overweight, (ii) very overweight, (iii) class I obese, (iv) class II obese, (v) class III obese	

**Table 6:** The subclasses and individual entries of food items class.

	Fourteen subclasses (14) of food items class	Individuals of each food items class
Thing → ingredients → food items	(1) Fishery marine products	∗
	(2) Meat	∗
	(3) Bakery products	∗
	(4) Drinks	∗
	(5) Processed food	∗
	(6) Fruits vegetables	∗
	(7) Food supplement	∗
	(8) Salts spices soups sauces salads protein products	Mustard, hydrolyzed vegetable protein, hydrolyzed protein, black pepper, bran, red pepper, whey protein, and so forth.
	(9) Salty spicy mild snacks food	Pistachio, almond, almond paste, walnut, nut, nut paste, and so forth.
	(10) Egg products	Powdered egg, egg, albumen, egg product, egg yolk, egg product, and so forth.
	(11) Sugars syrups honey sweeteners	Honey, fructose syrup, glycose syrup, glucose syrup, malt, malt extract, whey syrup sugar, and so forth.
	(12) Macro nutritional elements	Carbohydrates, proteins, water, dairy products analogous, cereals milled cereal products, sweetener agents aromas, desserts, oils and fats, and so forth.
	(13) Micro nutritional elements	Alanine, aspartic acid, water soluble fiber, and so forth.
	(14) Food items no. (FI)	^*^FI001–FI112

^*^Various individuals are declared in the subclasses of food items (FI). Food items class contains many subclasses (only 14 classes are depicted above) each with numerous individuals; therefore, Table 6 contains only a part of the individuals of these subclasses.

**Table 7:** The subclasses and individual entries of food additives class.

Main classes	Subclasses of food additives	Values (individuals)
Thing → ingredients → food additives	Codex no.	Codex numbers of almost 1500 different food additives are declared as individuals of the class.
	Food additive name	Almost 1500 different food additives names are declared as individuals of the class.
	Food additive type	Antioxidant, acidity regulators, emulsifiers, carbonating, gelling agent, bulking agent, carbonating agent, antifoaming agent, flavor enhancer, color retention agent, color, firming, carrier, flour treatment agent, forming agent, and so forth.
	E-codex no.	E-codex numbers of almost 1500 different food additives are declared as individuals of the class.

**Table 8:** The subclasses and individual entries of product class.

Main classes	Subclasses of the products	
Thing → products	Product category name	Bakery products, candies, cereals and cereals products, drinks, edible ices, egg and egg products, food for particular nutritional uses, fruit and vegetables, liquid and solid oils, liquid and solid oils, milk and milk products, ready to consume salty spicy light meals and snacks, salts spices soups sauces salad and protein products, seafood, sugars syrups honey and table sweeteners, and so forth.
	Product category no.	The above product type category names are numbered and added to this class.
	Product EAN	The barcodes or QR code numbers are inserted into the class instantly each run.

**Table 9:** OWL 2.0 syntax in food ontology knowledge base (FOKB) in the system.

<?xml version=“1.0”?> <Ontology xmlns=“http://www. w3. org/…/owl#” xml:base=“http://… /ont/FOKB” <Declaration> <Class IRI=“#Acidity_Regulator”/> </Declaration> <Declaration> <Class IRI=“#Anticaking_Agent”/> </Declaration> <Declaration> <Class IRI=“#Antioxidant”/> </Declaration> <Declaration> <Class IRI=“#Ascorbic_Acid”/> </Declaration> <Declaration> <Class IRI=“#D-Isoascorbic_Acid”/> </Declaration> <Declaration> <NamedIndividual IRI=“#Ascorbic_Acid”/> </Declaration> <ClassAssertion> <Class IRI=“#Ascorbic_Acid”/> <NamedIndividual IRI=“#Ascorbic_Acid”/> </ClassAssertion> <SubClassOf> <Class IRI=“#Ascorbic_Acid”/> <Class IRI=“#Antioxidant”/> </SubClassOf> <SubClassOf> <Class IRI=“#Ascorbyl_Palmitate”/> <Class IRI=“#Ascorbic_Acid”/> </SubClassOf>	<SubClassOf> <Class IRI=“#D-Isoascorbic_Acid”/> <Class IRI=“#Ascorbic_Acid”/> </SubClassOf> <SubClassOf> <Class IRI=“#Ester-C”/> <Class IRI=“#Ascorbic_Acid”/> </SubClassOf> <ObjectPropertyAssertion> <ObjectProperty IRI=“#Has_Deficiency_Disease”/> <NamedIndividual IRI=“#Ascorbic_Acid”/> <NamedIndividual IRI=“#Scurvy”/> </ObjectPropertyAssertion> <ObjectPropertyAssertion> <ObjectProperty IRI=“#Has_Overdose_Disease”/> <NamedIndividual IRI=“#Ascorbic_Acid”/> <NamedIndividual IRI=“#Vitamin_C_mega_dosage”/> </ObjectPropertyAssertion> <ObjectPropertyAssertion> <ObjectProperty IRI=“#Has_Synonym”/> <NamedIndividual IRI=“#Ascorbic_Acid”/> <NamedIndividual IRI=“#E300”/> </ObjectPropertyAssertion> <ObjectPropertyAssertion> <ObjectProperty IRI=“#Has_Synonym”/> <NamedIndividual IRI=“#Ascorbic_Acid”/> <NamedIndividual IRI=“#C_Vitamin”/> </ObjectPropertyAssertion> <ObjectPropertyAssertion> <ObjectProperty IRI=“#Has_Solubility”/> <NamedIndividual IRI=“#Ascorbic_Acid”/> <NamedIndividual IRI=“#Water”/> </ObjectPropertyAssertion> </Ontology>

**Table 10:** The model used while creating the subclasses and individual entries of some well-known vitamins in the FOKB.

Vitamin	Vitamin chemical name(s)	Solubility	Recommended dietary allowances	Deficiency disease	Upper intake level	Overdose disease
Vitamin A	Retinol, retinal, and four carotenoids including beta carotene	Fat	**900 *µ*g**	Night-blindness, hyperkeratosis, and keratomalacia	3,000 *µ*g	Hypervitaminosis A

Vitamin B_1_	Thiamine	Water	**1.2 mg**	Beriberi, Wernicke-Korsakoff syndrome	N/D	Drowsiness or muscle relaxation with large doses

Vitamin B_2_	Riboflavin	Water	**1.3 mg**	Ariboflavinosis, glossitis, and angular stomatitis	N/D	

Vitamin B_3_	Niacin, Niacinamide	Water	**16.0 mg**	Pellagra	35.0 mg	Liver damage (doses > 2 g/day) and other problems

Vitamin C	Ascorbic Acid	Water	**90.0 mg**	Scurvy	2,000 mg	Vitamin C mega dosage

**Table 11:** 

Consumer (*C*)	D-Isoascorbic Acid, Algin, and Vitamin B_3_
*P*_1_ ingredients	Sodium Alginate, Ascorbic Acid, and Folic Acid
*P*_2_ ingredients	Ascorbyl Palmitate, Niacin, and Alginic Acid

## Appendices

### A. Class Diagram of FOKB

For more details see Figure 9(a).

### B. Flow Diagram of FoodWiki Mobile System

For more details see Figure 9(b).

## References

[1] Berners-Lee T., Hendler J., Lassila O. (2001). The semantic web. *Scientific American*.

[2] Gruber T. (2007). *What Is an Ontology?*.

[3] W3C (2014). *OWL 2.0, Web Ontology Language Overview, W3C Recommendation*.

[4] Cantais J., Dominguez D., Gigante V., Laera L., Tamma V. An example of food ontology for diabetes control.

[5] American Diabetes Association (2007). Nutrition recommendations and interventions for diabetes: a position statement of the American Diabetes Association. *Diabetes Care*.

[6] Li H.-C., Ko W.-M. Automated food ontology construction mechanism for diabetes diet care.

[7] Koenderink N. J. J. P., Hulzebos L., Rijgersberg H., Top J. L. Food informatics: sharing food knowledge for research and development.

[8] Snae C., Brueckner M. (2007). Personal health assistance service expert system (PHASES). *Life Sciences*.

[9] Protégé OWL Ontology (2014). *Protégé 4. 1 Tool*.

[10] (2004). *SWRL: A Semantic Web Rule Language Combining OWL and RuleML*.

[11] Paolucci M., Kawamura T., Payne T. R., Sycara K. (2002). Semantic matching of web services capabilities. *Proceedings of the 1st International Semantic Web Conference on Semantic Matching of Web Services Capabilities, Sardinia, Italy, June 2002*.

[12] Bener A. B., Özadalı V., Ilhan E. S. A. (2009). Semantic matchmaker with precondition and effect matching using SWRL. *Expert Systems with Applications*.

[13] Ilhan E. S., Bener A. B. Improved service ranking and scoring: semantic advanced matchmaker (SAM) architecture.

[14] Ilhan E. S., Akkus G. B., Bener A. B. SAM: semantic advanced matchmaker.

[15] Çelik D., Elçi A. (2013). A broker-based semantic agent for discovering Semantic Web services through process similarity matching and equivalence considering quality of service. *Science China Information Sciences Journal*.

[16] Cort W. M. (1974). Antioxidant activity of tocopherols, ascorbyl palmitate, and ascorbic acid and their mode of action. *Journal of the American Oil Chemists' Society*.

[17] Austria R., Semenzato A., Bettero A. (1997). Stability of vitamin C derivatives in solution and topical formulations. *Journal of Pharmaceutical and Biomedical Analysis*.

[18] Sauberlich H. E., Tamura T., Craig C. B., Freeberg L. E., Liu T. (1996). Effects of erythorbic acid on vitamin C metabolism in young women. *The American Journal of Clinical Nutrition*.

[19] Winkler W. E. (1999). The state of record linkage and current research problems.

[20] Winkler W. E., Thibaudeau Y. (1991). An application of the Fellegi-Sunter model of record linkage to the 1990 U.S. decennial census. *Statistical Research Report Series*.

[21] OWL API (for OWL 2.0) http://owlapi.sourceforge.net/.

[22] Sirin E., Pellet P. B. An OWL DL reasoner.

[23] Çelik D., Elçi A., Akçiçek R., Gökçe B., Hürcan P. A safety food consumption mobile system through semantic web technology.

